# Editorial: Advancements in molecular and cellular mechanisms of stem cells in tissue development and regeneration

**DOI:** 10.3389/fcell.2026.1919782

**Published:** 2026-07-14

**Authors:** Ajoy Aloysius, Vishnu Vijay Vijayan, Sumanth Manohar, Finosh G. Thankam

**Affiliations:** 1 Department of Biology, University of Kentucky, Lexington, KY, United States; 2 Department of Pathology, Dalhousie University, Halifax, NS, Canada; 3 Beatrice Hunter Cancer Research Institute, Halifax, NS, Canada; 4 Department of Translational Research, College of Osteopathic Medicine of the Pacific, Western University of Health Sciences, Pomona, CA, United States

**Keywords:** cell cell communication, development, regenerative therapy, stem cell (SC), tissue engineeering, tissue regeneration

## Introduction

Stem cells are fundamental to embryonic development, tissue homeostasis, and regenerative repair following injury. Their unique capacity for self-renewal and differentiation into diverse cell types has positioned stem cell research at the forefront of regenerative medicine. Over the past decade, significant advances have enhanced our understanding regarding the molecular mechanisms governing stem cell activation, proliferation, differentiation, and plasticity, as well as their communication with neighboring cells and the surrounding microenvironment during development and tissue repair.

These seminal discoveries have opened new avenues for therapeutic interventions in neurodegenerative diseases, metabolic diseases, traumatic injuries, developmental disorders, and age-associated tissue degeneration (De Luca et al., 2019). Furthermore, recent advancements in stem cell engineering, transplantation, and biomaterial technologies foster the development of personalized regenerative therapies, with the potential to improve patient outcomes and quality of life.

This research topic brings together studies that investigate the cellular and molecular mechanisms underlying stem cell identity and plasticity, the microenvironmental cues that shape tissue development and regeneration, and translational strategies aimed at harnessing stem cells for therapeutic applications. Collectively, these contributions underscore the importance of understanding fundamental stem cell biology as a prerequisite for developing effective regenerative therapies. The studies span basic, mechanistic, and translational research, highlighting the critical role of versatile stem cells in determining regenerative outcomes.

## Tissue development and homeostasis

Stem cell fate decisions within progenitor populations are essential for tissue development and homeostasis. Using three-dimensional brain organoids derived from pluripotent stem cells, Alonso-Olivares et al. investigated the molecular mechanisms governing cortical development and specifically examined the roles of p73 and p53 in neuronal progenitors during mouse brain development. Their findings identified p73 as a key regulator of neural cell fate determination. To advance the study of neurodevelopmental disorders, Kelkar et al. developed a human induced pluripotent stem cell (iPSC) UBE3A fluorescent reporter line as a tool to investigate Angelman syndrome. This innovative model provides a valuable platform for investigating UBE3A function and for screening potential therapeutic compounds. Feng et al. examined the role of miR-185-5p, a highly enriched microRNA in embryonic neural progenitors, during brain development. This study demonstrated that miR-185-5p plays a critical role in maintaining the balance between progenitor proliferation and neuronal differentiation, thereby ensuring proper neural development. These studies provide a fascinating glimpse into the intricate mechanisms behind stem cell tissue development, deepening our understanding of how these remarkable cells contribute to growth and regeneration.

## Tissue repair and regeneration

Stem cells contribute to both physiological tissue regeneration and pathological tissue remodeling; however, the mechanisms distinguishing these processes remain incompletely understood. Using long-term *ex vivo* cultures of normal endometrium and uterine fibroids, Vázquez et al. performed comprehensive transcriptomic analyses that revealed alterations in multiple cellular pathways associated with stem cell function. The findings provided insights into fibroid pathogenesis and identified potential therapeutic targets. Activation and differentiation of stem cells are essential for tissue repair following injury, yet many of the underlying regulatory signals remain poorly defined. Wu et al. identified a previously unrecognized role for the sensory nerve-derived neuropeptide calcitonin gene-related peptide (CGRP) in dental pulp regeneration. Beyond stimulating stem cell activation, CGRP coordinated vascularization, highlighting the importance of neurovascular-stromal interactions during tissue repair. Using genetic and pharmacological approaches, Raghavan et al. demonstrated the diverse and context-dependent roles of microglia during retinal injury and repair. Their findings suggest a much more nuanced role for microglia during photoreceptor degeneration and a targeted modulation of microglial activity may represent a promising strategy for enhancing retinal regeneration. In a brief research report, Yu et al. uncovered a novel function of the lysosomal gene *Cln3*, mutations of which cause Batten disease (a rare, fatal, inherited neurological disorder). Using *Drosophila* models, the study demonstrated that Cln3 is required for intestinal stem cell activation following injury through regulation of JAK/STAT signaling. Aging is a major contributor to stem cell dysfunction and impaired tissue regeneration. Zhao et al. reported that kaempferol, a naturally occurring flavonoid, promotes intestinal stem cell homeostasis, enhances tissue repair, and extends lifespan in aged *Drosophila*. This study suggests that the antioxidant properties of kaempferol may have therapeutic potential to combat the age-related decline in regenerative capacity. Together, these findings underscore the significance of stem cells in adult tissue regeneration and show that their molecular alterations lead to the repair outcome.

## Cellular communication in stem cell function

Stem cells continuously communicate with their microenvironment through diverse signaling modalities that regulate development, homeostasis, and regeneration. Jagdish et al. investigated cell-fate transitions in heterogeneous embryonic stem cell populations using computational approaches to reconstruct cell-to-cell communication networks. Their analysis highlighted the importance of paracrine signaling during early developmental lineage decisions. Sun et al. explored how aging-associated alterations in the stem cell niche contribute to dental pulp fibrosis. Their study revealed that specific macrophage populations accumulate during aging and interact with dental pulp progenitor cells, creating a pro-inflammatory microenvironment that promotes fibrosis and compromises stem cell function. Estrada Elorza et al. provided a comprehensive review of mechanotransduction across cellular, transcriptional, and tissue levels. Notably, they proposed the intriguing hypothesis that certain mechanosensors may be dispensable during embryonic morphogenesis; however, is essential during tissue repair. This concept offers an exciting framework for future studies and have important implications for regenerative medicine.

## Stem cell therapy for disease

Despite significant advancements in our understanding of stem cell biology, translating these discoveries into effective clinical therapies remains a considerable challenge. One of the major unmet needs in osteoarthritis treatment is cartilage regeneration. Wei et al. conducted a systematic evaluation of the chondrogenic potential of various stem cell populations cultured within scaffolds coated with different decellularized extracellular matrix (dECM) preparations. Their optimized strategy demonstrates considerable promise for cartilage repair and regenerative treatment of osteoarthritis.

Several studies in this collection investigated stem cell-based interventions for pregnancy-associated deep vein thrombosis (DVT), a significant cause of maternal morbidity and mortality. Using a rat model, Zhang et al. performed multi-omics analyses demonstrating that umbilical cord-derived mesenchymal stem cells (UC-MSCs) possess substantial therapeutic potential in pregnancy-associated DVT. Complementing these findings, Xie et al. demonstrated that transplantation of bone marrow-derived mesenchymal stem cells (BM-MSCs) improved pregnancy outcomes in DVT models by restoring the balance between pro- and anti-angiogenic factors, thereby enhancing placental perfusion. Together, these studies highlight the therapeutic potential of MSC-based interventions for vascular complications during pregnancy. Using a radiation-induced muscle fibrosis model, Li et al. demonstrated that adipose-derived stem cells (ADSCs) exert protective effects by promoting muscle satellite cell activation and differentiation. Their findings support ADSCs as promising candidates for the treatment of muscle fibrosis and regenerative disorders.

Two comprehensive reviews further emphasize the therapeutic potential of stem cells. Li et al. synthesized current knowledge regarding the pathogenesis of degenerative disc disease and critically evaluated emerging MSC-based regenerative strategies. Matiukhova et al. reviewed the rapidly expanding applications of induced pluripotent stem cells (iPSCs) in biomedical research and regenerative medicine, providing an extensive overview of current methodologies, disease modeling approaches, and therapeutic opportunities.

## Conclusion

Collectively, the studies presented in this research topic advance three interconnected concepts. First, regeneration is an emergent process arising from the integration of intrinsic genetic programs and extrinsic niche-derived signals. Second, regenerative outcomes are highly context dependent; identical signaling pathways could promote tissue repair or drive pathological remodeling depending on cellular state, timing, and microenvironmental conditions. Third, integrative approaches that combine multi-omics technologies, advanced model systems, and functional analyses are accelerating translation by linking mechanistic insights to therapeutic applications and identifying biomarkers of efficacy and safety.

Importantly, the findings highlighted in this collection suggest that successful regenerative therapies warrant strategies that extend beyond simple stem cell replacement. Future interventions are likely to involve coordinated modulation of signaling networks, immune and vascular niches, and biomaterial scaffolds that support tissue integration and functional recovery. Approaches such as ECM-based preconditioning, engineered biomaterials that promote vascularization, and exosome-based or combinatorial therapies emerge as particularly promising directions for the next-generation regenerative medicine.
